# The psychological impact of the COVID-19 pandemic on adults with autism: a survey study across three countries

**DOI:** 10.1186/s13229-021-00424-y

**Published:** 2021-03-03

**Authors:** Danna Oomen, Annabel D. Nijhof, Jan R. Wiersema

**Affiliations:** 1 Department of Experimental Clinical and Health Psychology, Ghent University, Henri Dunantlaan 2, 9000 Ghent, Belgium; 2 EXPLORA, Ghent University, Ghent, Belgium

**Keywords:** COVID-19, Pandemic, Autism, Mental health

## Abstract

**Background:**

Previous studies have reported a negative psychological and mental health impact of the COVID-19 pandemic. This impact is likely to be stronger for people with autism as they are at heightened risk of mental health problems and because the pandemic directly affects social functioning and everyday routines. We therefore examined COVID-19 pandemic-related changes in mental health, the impact of the pandemic on their social life and routines, satisfaction with pandemic-related information and tips, and participants’ wishes for guidance.

**Methods:**

We used a mixed-method approach, collecting quantitative and qualitative survey data from adults with and without autism across three European countries: Belgium, the Netherlands, and the UK (*N* = 1044).

**Results:**

We found an increase in depression and anxiety symptoms in response to the pandemic for both the non-autism and the autism group, which was greater for adults with autism. Furthermore, adults with autism showed a greater increase in worries about their pets, work, getting medication and food, and their own safety/security. They felt more relieved from social stress, yet experienced the loss of social contact as difficult. Adults with autism also felt more stressed about the loss of routines. Pleasant changes noted by adults with autism were the increase in solidarity and reduced sensory and social overload. Adults with autism frequently reported problems with cancellation of guidance due to the pandemic and expressed their wish for (more) autism-specific information and advice.

**Limitations:**

Our sample is likely to reflect some degree of selection bias, and longitudinal studies are needed to determine long-term effects.

**Conclusions:**

Results highlight the psychological burden of the pandemic on adults with autism and shed light on how to support them during this COVID-19 pandemic, which is especially important now that the pandemic is likely to have a prolonged course. There is a need for accessible, affordable (continued) support from health services. Guidance may focus on the maintenance of a social network, and adjusting routines to the rapid ongoing changes. Finally, we may learn from the COVID-19 pandemic-related changes experienced as pleasant by adults with autism to build a more autism-friendly society post-pandemic.

## Introduction

On March 11, 2020, the World Health Organization (WHO) officially declared the COVID-19 virus a global pandemic [1]. The global spread of the virus led to a massive and immediate public health campaign to reduce further spread. This campaign encouraged large-scale behavior change, including social isolation and distancing, which has a massive impact on how we live and socialize on a daily basis. Added to these many daily life changes (e.g., studying/working from home, loss of activities, and real-life social contact) that we had to adapt to rapidly, this unprecedented pandemic comes with great uncertainty and heightened levels of threat (e.g., worry about loved ones or yourself contracting the virus; [2]). Both the pandemic itself and the encouraged behavior change can have an impact on our mental health as uncertainty is a cognitive and psychological stressor [3], while social isolation has consistently been associated with negative mental health outcomes and conversely, social support is considered an important buffer in adverse times (e.g., [4]). Hence, concerns have been raised regarding the impact of the pandemic and the associated containment measures on the mental health of individuals across society (e.g., [5]). Studies on previous epidemics as well as the current COVID-19 pandemic indeed highlight their negative mental health impact (e.g., Equine influenza: [6]; SARS: [7, 8]; H1N1: [9]; COVID-19: [10–16]).

Importantly, although the COVID-19 pandemic has an influence on society as a whole, negative mental health effects due to the pandemic might not be evenly distributed. Individuals with autism spectrum disorder (ASD; henceforth ‘autism’^1^) may be considered one of the vulnerable groups [5] that are likely to be affected to a greater extent. Autism is a prevalent neurodevelopmental disorder with a global worldwide prevalence estimated as 0.62% or higher [17]. The impact of the pandemic may be stronger for individuals with autism for two main reasons: Firstly, autism is associated with a heightened risk of mental health problems, including a higher likelihood of comorbid mental health disorders such as mood and anxiety disorders [18, 19]. Secondly, autism is characterized by two core symptom domains which are directly impacted by the pandemic. On the one hand, individuals with autism experience social interaction and communication difficulties, such as difficulties initiating or responding to social interactions, adjusting their behavior to suit various social contexts, and developing and maintaining relationships. On the other hand, individuals with autism show repetitive and restricted behavior, interests, and activities. The latter domain includes insistence on sameness and inflexible adherence to routines, which sometimes results in extreme distress in response to small (routine) changes and difficulties with transitions. These two core symptom domains of autism are directly affected by the pandemic, as it both impacts our social interactions and results in big, continuing changes to our everyday lives. This gives reason to believe that individuals with autism may experience the pandemic in a unique way. Given the fact that adults with autism are reported to have more difficulty coping with stressful events than neurotypicals do [20, 21], and especially because levels of anxiety and depression are known to be generally high in the autism population [18, 19], there is a need for research to ascertain the impact of the pandemic on the mental health of individuals with autism.

This need for research on COVID-19 and autism is also evident from the emerging articles that emphasize the concerns for individuals with autism during the COVID-19 pandemic [22–29]. Asbury et al. [23] asked parents of school-aged children with special educational needs and disabilities to describe the impact of the COVID-19 pandemic on the mental health of their child. The majority of the sample consisted of parents of a child with autism (82%). Children were reported to experience anxiety/worry and low mood. In the study of Amorim et al. [26], caregivers of children with autism also reported more anxiety during quarantine, both for themselves and their child, compared to children without autism and their caregivers. Furthermore, the majority of caregivers in the autism group reported that quarantine negatively impacted emotion management in their children, whereas the majority of parents without autism reported no impact or even a positive impact of quarantine on emotion management in their children. Studies on behavioral problems during the COVID-19 pandemic have found an increase in behavioral problems in children with autism since the pandemic [28], and that preexisting behavioral problems predict a higher risk of more intense and frequent behavioral problems during the pandemic [29]. The commentary of Ameis et al. [22] highlights the potential negative impact of changes to everyday routines and restrictions to regular services on children with autism. White et al. [24] surveyed caregivers of children with autism and indeed found services and therapies for individuals with autism to be disrupted. Thus far, there is only one survey that most likely reports data from adults (among younger responders): the National Autistic Society, a charity, published a report [27] which revealed that 9 out of 10 people with autism worried about their mental health during lockdown. However, they did not include a control group or sample characteristics, preventing any conclusions about how these mental health worries compare to the general population, or about how many of the respondents were adults. To date, no study has systematically investigated the psychological impact of the COVID-19 pandemic on adults with autism specifically. The current study therefore focuses on adults with autism, and includes a control group of adults without autism. Information given directly by adults with autism themselves will provide valuable insights into the influences of social distancing measures and abrupt changes to routines on the mental health and lives of adults with autism. These insights will help to form actions to mitigate the negative effects of the COVID-19 pandemic. Furthermore, insights may be relevant for future pandemics and/or public health crises.

### Current study

The current study aimed to ascertain the impact of the COVID-19 pandemic and the associated containment measures on the psychological well-being of adults with autism (in comparison with adults without autism), as well as their needs throughout this time. As the COVID-19 pandemic affects people globally, we sampled broadly and included participants from three European countries: Belgium, the Netherlands, and the UK.^2^ The governments of each of these three countries released a statement to self-isolate or social distance halfway through March (i.e., Belgium: 18 March, the Netherlands: 15 March, UK: 23 March), and Belgium started to relax its containment measures in the beginning of May. The current study focused on the beginning of the pandemic and containment measures until the first relaxation of these measures. Data collection ran from April 3 to May 7, 2020.

The main focus was to investigate the impact of the pandemic and the associated containment measures on the mental health of adults with autism by assessing depression and anxiety symptoms and specific worries. We also assessed how the pandemic affected the two autism symptom domains by inquiring about the impact on their social life (domain I), and their everyday life and routines (domain II). In addition, we asked the participants how satisfied they are with the pandemic-related information and tips available, and whether people received guidance or had a wish for guidance during this pandemic. We applied both a quantitative and qualitative approach. Qualitative data were gathered to gain a broader insight into individual needs, and to explain what pandemic-related changes were experienced as the most difficult, caused the most stress/anxiety, and made life more pleasant (if any).

To the best of our knowledge, this is the first systematic study on the psychological impact of the pandemic on adults with autism. For this reason, we had no strong specific hypotheses. We did however expect an overall negative mental-health impact across groups, in line with previous research in neurotypical populations (e.g., [11]). Furthermore, we expected the negative mental health impact of the pandemic overall to be greater for adults with autism than adults without autism.

## Methods

### Participants and procedure

The survey was completed by 1044 participants (*n*_female_ = 718, 68.8%; *n*_male_ = 310, 29.7%; *n*_other_ = 16), of whom 613 (58.7%) indicated to have a formal clinical diagnosis of autism.^3^ Just under half of the sample were residents of Belgium (*n*_Belgium_ = 517, 49.5%; *n*_the Netherlands_ = 244, 23.4%; *n*_UK_ = 283, 27.1%). Age ranged from 18 to 78 years old for the autism group (*M* = 38.36, SD = 11.59) and from 18 to 81 for the non-autism group (*M* = 38.38, SD = 14.14). Mean age did not differ between groups, *t*(805.91) = − 0.024. There were also no group differences in gender ratio, *χ*^2^(1) = 1.43, *p* = 0.232 or in years of education, *t*(1042) = − 1.12, *p* = 0.263. See Table 1 for all demographic data.

**Table 1:** Demographic data per group

	Autism *M* (SD)/*n* (%)	Non-autism *M* (SD)/*n* (%)
Age	38.36 (11.59)	38.38 (14.14)
Sex (female)^a^	409 (68.4%)	309 (71.86%)
Years of education	16.77 (4.98)	17.12 (4.77)
Employment status		
Student	57 (9.3%)	53 (12.3%)
Self-employed	28 (4.6%)	35 (8.1%)
Employed (part- and full-time)	278 (45.3%)	265 (61.4%)
Unable to work due to disability	164 (26.8%)	26 (6.0%)
Homemaker	20 (3.3%)	3 (0.7%)
Seeking work	44 (7.2%)	17 (3.9%)
Retired	22 (3.6%)	32 (7.4%)
Country of residence		
Belgium	305 (49.8%)	212 (49.2%)
The Netherlands	124 (20.2%)	120 (27.8%)
The UK	184 (30.0%)	99 (23.0%)
AQ-short	86.03 (11.79)	58.99 (13.87)
Social behavior	72.18 (10.11)	48.74 (12.19)
Social skills	22.46 (4.09)	14.84 (5.31)
Routine	13.12 (2.24)	9.24 (2.88)
Switching	13.55 (2.16)	9.08 (2.90)
Imagination	23.05 (4.78)	15.58 (4.19)
Numbers and patterns	13.85 (3.59)	10.26 (3.71)
Other diagnoses^b^		
ADHD	93 (15.2%)	19 (4.4%)
Mood disorder	210 (34.3%)	44 (10.2%)
Anxiety disorder	158 (25.8%)	36 (8.4%)
Other	79 (12.9%)	11 (2.6%)
None	261 (42.6%)	355 (82.4%)

Autism group: *n* = 613, non-autism group: *n* = 431

^a^3 cases that indicated intersex (autism group *n* = 3; non-autism group *n* = 0) and 13 cases that indicated ‘prefer not to say’ (autism group *n* = 12; non-autism group *n* = 1) are not included in the percentages of sex, due to an insufficient number of cases

^b^ Following the DSM-5 (e.g. ADHD includes ADD). Examples of ‘other’ category: trauma and stress-related, obsessive–compulsive, psychotic, eating, personality, learning disorders

Participants were recruited via online advertisement through various social media channels, organizations (e.g., discover network of Autistica) and via existing databases of individuals with autism that have previously given their consent to be contacted for autism-related research. Participants had to be 18 years or older, residents of Belgium, the Netherlands, or the UK and have sufficient knowledge of the English language. Following an online information letter and consent form, participants were asked to fill out a series of short questions assessing their personal situation, their pandemic experience, autism symptomatology, and the impact of the pandemic on their daily life and mental health. The survey took approximately 20 min to fill out. On every page of the survey participants were provided with links to resources for (mental health) support and pandemic-related information in case they needed them. This study was approved by the local ethics committee of the Faculty of Psychology and Educational Sciences of Ghent University (EC/2020/46).

### Survey

See “Appendix A” for the full questionnaire. Before going online, the survey was checked by an adult with autism who provided feedback on (the clarity of) the questions.

#### Demographics

We inquired about the following demographics: age, biological sex, country of residence, years of education (starting from year 1, i.e. learning to read), own employment status, and (if applicable) their partner’s employment status.

#### Personal situation

We inquired about participants’ personal situation by assessing their living situation (e.g. assisted living facility, living with flat mates), and the number of adults, children, and pets living with them.

#### Personal situation: COVID-19

We inquired about the COVID-19 specific personal situation of participants by assessing the following topics: Whether they have (had) COVID-19; time spent actively looking up/reading about COVID-19; time spent going over COVID-19-related information in their minds; their self-perceived COVID-19 knowledge level; how strictly they think they are following the recommendations from national authorities; how the pandemic influenced their daily routines (e.g., living as normal, self-isolation, social distancing); usual amount spent socially interacting; and the number of days they: self-isolated, went outside, met someone outside, had face-to-face contact, had a phone or video call.

#### Autism spectrum disorder

We inquired whether participants had a formal clinical diagnosis of autism or not. To ensure answers were reliable, questions were added inquiring about the specific diagnosis, and the age of diagnosis. Additionally, the survey also included a question inquiring about other formal clinical diagnoses (ADHD, depressive or anxiety disorder, or other). Autism symptomatology was assessed with the Autism spectrum quotient-short (AQ-short; [30]) in all participants. The AQ-short contains 28 items comprising two higher-order factors: ‘social behavioral difficulties’ and ‘fascination for numbers/patterns’. The former factor can further be divided into four lower-order factors: ‘social skills’, ‘routine’, ‘switching’ and ‘imagination’. Items were rated on a four-point scale ranging from 1 (‘definitely agree’) to 4 (‘definitely disagree’; total score ranging from 28 to 112). Reverse items are included in such a way that agreement suggests the presence of an autistic-like trait in half of the items. Hoekstra et al. [30] suggest a cut-off of > 65. The internal consistencies of the AQ-short in the current sample were excellent (Total: *α* = 0.94; Social behavior: *α* = 0.94; Social skills: *α* = 0.91) and good (Routine: *α* = 0.82; Switching: *α* = 0.82; Imagination: *α* = 0.84; Numbers and patterns: *α* = 0.81).

#### Depression and anxiety

To asses COVID-19 pandemic-related changes in depression symptoms we adapted the Patient Health Questionnaire-9 (PHQ-9; [31]). The PHQ-9 contains 9 items designed to correspond to the nine DSM-IV diagnostic criteria for major depressive disorder, and an impact question (i.e., “How difficult have these problems made it for you to do your work, take care of things at home, or get along with other people?”). To assess COVID-19 pandemic-related anxiety-symptom changes we adapted the Generalized Anxiety Disorder scale (GAD-7; [32]). The GAD-7 contains 7 items designed in part on the basis of the DSM-IV criteria for GAD, and an impact question. All items of both screeners were included, but to capture COVID-19 pandemic-related changes in depression and anxiety symptomatology we adjusted the rating scale: 1 (‘significantly less than usual’), 2 (‘slightly less than usual’), 3 (‘not more than usual’), 4 (‘slightly more than usual’), 5 (‘significantly more than usual’). We left the four-point scale of the impact questions unchanged, ranging from 1 (‘not difficult at all’) to 4 (‘extremely difficult’). In this paper, we will refer to the *adapted* PHQ-9 and GAD-7. The internal consistency of the *adapted* PHQ-9 was good, *α* = 0.88. The internal consistency of the *adapted* PHQ-9 was excellent, *α* = 0.91.

#### Worries

We inquired whether in the last two weeks, participants experienced more or less worries than usual about their marriage or other romantic relationship(s), pet(s), access to medication, food and internet, safety/security, future plans, finances, work (even if they feel their job is safe), losing their job, and boredom. Items were rated on a five-point scale: 1 (‘significantly less than usual’), 2 (‘slightly less than usual’), 3 (‘not more than usual/ not applicable’), 4 (‘slightly more than usual’), 5 (‘significantly more than usual’). Furthermore, we inquired whether participants experienced the following COVID-19 pandemic specific worries: following the recommendations to prevent spread of COVID-19 correctly, catching COVID-19, and friends or family catching COVID-19. Items were rated on a five-point scale ranging from 1 (‘not at all’) to 5 (‘very much so’).

#### Social life and routines

We assessed the impact of the pandemic on the two symptom domains of autism through the following three questions on their social life and routines. Social life: (1) To what extent did the COVID-19 pandemic change your social life?; (2) Due to the effects of the COVID-19 regulations on my everyday life, I feel socially isolated, and (3) Due to the effects of the COVID-19 regulations on my everyday life, I feel relieved from social stress. Routines: (1) To what extent do you feel you have/ had to adjust your daily routines due to the COVID-19 pandemic?; (2) Due to the effects of the COVID-19 regulations on my everyday life, I feel stressed about changes to my daily routines, and (3) Due to the effects of the COVID-19 regulations on my everyday life, I enjoy the freedom to deviate from society’s expectations and to adjust my routines to my personal preference. All six items were rated on a five-point scale ranging from 1 (‘not at all’) to 5 (‘very much so’).

#### Guidance

We asked whether participants received any help/guidance from a qualified medical specialist (e.g., psychiatrist, psychologist, counsellor) in the last 6 months, and if they answered ‘no, but I would like to’, whether their wish for help/guidance was related or unrelated to the pandemic. Furthermore, we assessed how satisfied they were with the tips and tools that are available during the COVID-19 pandemic. If someone indicated to have a formal diagnosis of autism, we adjusted this question to include ‘for individuals with autism’. Lastly, participants with autism were also asked how important it was to them that individuals with autism are consulted for the development of these autism-specific tips and tools. These guidance questions were not mandatory.

#### Qualitative questions

Four (non-obligatory) open questions inquired about: the particular needs people have in order to manage their mental health during the current COVID-19 pandemic, as well as the COVID-19 pandemic-related changes on everyday life that were the most difficult, that have caused the most stress/anxiety, and that have made life more pleasant. These open questions could be filled in in either English or Dutch and were not mandatory.

### Data analysis

#### Quantitative data

Means with standard deviations or, where applicable, frequencies with percentages are reported for all demographics per group. We used linear mixed-effect models (LMMs) to detect group differences (autism versus non-autism group) on our outcome variables: COVID-19 pandemic-related changes in depression (*adapted* PHQ-9 total score) and anxiety symptoms (*adapted* GAD-7 total score), as well as on the additional question to assess the impact of the depression and anxiety symptoms on everyday life; 14 topics of worry (11 general ones, and 3 COVID-19 pandemic specific worries); the 6 routine (domain I) and social contact (domain II) questions (3 for each domain). In our models we included group as a fixed factor to detect group differences on our outcome variables. To control for variation coming from the three different countries, we included country as a (nested) random (slope and intercept) factor. LMMs allow us to take advantage of the full sample (power) and yet account for the correlation between data (i.e., non-independence) coming from the three different countries. This way we acquired results on the impact of the pandemic on adults that generalize across the three countries. We used the lme4 package to test the LMMs [33]. Lastly, for the questions on guidance, we reported frequencies with percentages.

In total we ran 24 LMMs. As a Bonferroni correction was regarded as too conservative, we made use of the False Discovery Rate (FDR) approach to correct for multiple testing [34]. In short, *q* values (FDR adjusted *p*-values) control the number of type I errors in the tests that result in a significant result, when determining adjusted *p*-values for each test. In the current study, we rejected 15 (62.5%) null hypotheses *w*ith q* = 0.05. Simply put, we estimate that 95% of these 15 rejections are true discoveries (following a Bayesian interpretation of the *q*-values; see [35]). Our interpretations will be based solely on the *q*-values, but for transparency we also report *p*-values. Note that Bonferroni correction for *p*-values would test each individual hypothesis at *α* = 0.05/24 = 0.002.

#### Qualitative data

In total, 82% of participants provided a response to at least one of the open questions (85% of the autism group, 78% of the non-autism group). Thematic analysis [36] was conducted by DO and AN. Both researchers independently familiarized themselves with the data by reading through the answers of each question of the first 200 participants, and noting down initial codes that described the key messages conveyed with the answers. We used an inductive approach for each open question, allowing the data to determine the codes. Thereafter the researchers met to discuss reoccurring codes to create preliminary codebooks—a separate codebook for each question—and to align the usage of code labels. After this, the four questions for all participants were independently coded by the two researchers separately. While coding, the researchers met regularly when the codebooks needed adjustment (e.g., breaking down codes into separate codes to obtain more specificity, inserting new codes that are not covered by an existing code). When all data were coded, the authors collaborated in identifying the most important codes per question (i.e., the ones that occurred the most often). We then generated themes that overarched the most important codes that related to each other. Codes were omitted that did not occur frequently or that did not significantly add to the developing themes. Lastly, we looked at how the themes fit together to describe the data in a coherent fashion, and whether they were autonomous, or subthemes. Themes, and where applicable subthemes, are presented together with example quotes to add in-depth meaning to the (sub)themes. We mostly selected quotes written in English, so as to avoid having them lost in translation, but quotes represent answers of the whole autism group. Quotes that we did translate from Dutch are indicated with “[translated]”. Results are reported per question, although some themes occurred under multiple questions, and were later assigned under the most appropriate question by the researchers to avoid too much repetition.

## Results

### Demographics

Demographic information on participants per group can be found in Table 1. The majority of adults with autism in our sample were female (68%), which is in disproportion with diagnostic rates [37], but common in large online studies (e.g., [38–40]). The larger part of the participants were residents of Belgium. Years of education between groups was similar, but relatively more adults without autism in our sample were employed (61% versus 45%; including self-employment: 70% versus 50%), in line with general findings of reduced employment in adults with autism [41, 42]. AQ-short scores within the autism group were comparable to previous samples [30, 39], as were the scores of the non-autism group [30]. Over half of the adults with autism in our sample had one or more comorbid psychiatric diagnoses (57%), whereas 18% of the adults without autism reported to be diagnosed with a mental health problem. We collected more information on pandemic-specific characteristics of our sample, as can be found in “Appendix B” Table 10. Not only did we inquire about participants’ COVID-19 pandemic-related behavior, we also asked extra questions regarding their specific living situation during the start of the pandemic.

### Quantitative analyses

#### Impact of the pandemic on depression and anxiety symptoms

Mean scores and standard deviations for the *adapted* PHQ-9 and GAD-7 can be found in Table 2. Across the two groups, three-quarters reported an increase in depression and anxiety symptoms in response to the pandemic (depression: 74%, anxiety: 75%, mean total score > 3). Our LMMs^4^ revealed that this increase in depression and anxiety symptoms was significantly greater in the autism group (total *adapted* PHQ-9 score: *β* = − 0.40, *χ*^2^ (1) = 11.86, *p* = 0.001, *q* = 0.001; total *adapted* GAD-7 score: *β* = − 0.32, *χ*^2^ (1) = 4.81, *p* = 0.028, *q* = 0.048). Importantly, the impact of the symptoms on everyday life was also greater in the autism group than the non-autism group (item 10 of the *adapted* PHQ-9: *β* = − 0.66, *χ*^2^ (1) = 76.20, *p* < 0.001, *q* < 0.001; item 8 of the *adapted* GAD-7: *β* = − 0.66, *χ*^2^ (1) = 59.27, *p* < 0.001, *q* < 0.001).

**Table 2:** COVID-19 pandemic-related change in depression and anxiety symptoms

	Autism *M* (SD)	Non-autism *M* (SD)
*Depression symptoms*		
Total *adapted* PHQ-9 score	3.57 (0.71)	3.30 (0.56)
Little interest or pleasure doing things	3.41 (1.06)	3.27 (0.86)
Feeling down, depressed, or hopeless	3.70 (1.07)	3.42 (0.89)
Trouble falling or staying asleep or too much sleep	3.86 (1.02)	3.51 (0.94)
Feeling tired or having little energy	3.79 (1.03)	3.46 (0.96)
Poor appetite or overeating	3.71 (0.91)	3.46 (0.80)
Feeling bad about yourself—or that you are a failure or have let yourself or your family down	3.54 (1.05)	3.31 (0.81)
Trouble concentrating on things, such as reading the newspaper or watching television	3.59 (1.00)	3.26 (0.79)
Moving or speaking so slowly that other people could have noticed	3.26 (0.77)	3.01 (0.56)
Thought that you would be better off dead, or of hurting yourself	3.22 (0.95)	2.97 (0.70)
Impact: How difficult have these problems made it for you to do your work, take care of things at home, or get along with other people^a^	2.39 (0.89)	1.82 (0.73)
*Anxiety symptoms*		
Total *adapted* GAD-7 score	3.62 (0.82)	3.38 (0.60)
Feeling nervous, anxious, or on edge	3.77 (1.06)	3.58 (0.87)
Not being able to stop or control worrying	3.63 (1.00)	3.40 (0.78)
Worrying too much about different things	3.67 (1.01)	3.41 (0.75)
Trouble relaxing	3.66 (1.03)	3.39 (0.87)
Being so restless that it’s hard to sit still	3.31 (0.91)	3.06 (0.64)
Becoming easily annoyed or irritable	3.64 (1.05)	3.46 (0.87)
Feeling afraid as if something awful might happen	3.63 (0.97)	3.39 (0.75)
Impact: How difficult have these problems made it for you to do your work, take care of things at home, or get along with other people?^a^	2.39 (0.88)	1.85 (0.69)

Autism group: *n* = 613, non-autism group: *n* = 431; Questions were rated on a five-point scale going from 1 (‘significantly less than usual’) to 5 (‘significantly more than usual’), except for the additional impact questions that were rated on a 4 point rating scale going from 1 (‘not difficult at all’) to 4 (‘extremely difficult’)

^a^Only includes participants that indicated 'slightly/significantly more than usual' to any of the above items. *Adapted* PHQ-9: autism group *n* = 524, non-autism group *n* = 340; *adapted* GAD-7: autism group *n* = 504, non-autism group *n* = 330

#### Pandemic-related changes in worries

Means, standard deviations, and LMM statistics of each of the worry topics are given in Table 3. On average, both groups indicated to worry more than usual about most topics of worry (mean scores > 3). Moreover, this increase in worries was significantly greater in the autism group for the following worry topics: Their pets, work (even when they felt their job was safe), losing their job, getting medication and food, and their own safety/security. The autism group did not worry more than the non-autism group about any of the COVID-19 pandemic specific worries.

**Table 3:** Mean scores per group regarding eleven general, and three pandemic-related topics of worry

	Autism *M* (SD)	Non-autism *M* (SD)	LMM statistics
Over the past 2 weeks, have you been more or less worried about the following:			
Marriage or other romantic relationship(s)	3.22 (0.90)	3.11 (0.86)	*β* = − 0.12, *χ*^2^ (1) = 1.33, *p* = .248, *q* = .313
Your pet^a^	3.25 (0.84)	2.99 (0.76)	*β* = − 0.32, *χ*^2^ (1) = 13.62, *p* < .001, *q* = .001
Work (even if you feel your job is safe)^b^	3.91 (0.96)	3.60 (1.03)	*β* = − 0.31, *χ*^2^ (1) = 14.33, *p* < .001, *q* = .001
Losing your job^b^	3.52 (1.04)	3.24 (0.99)	*β* = − 0.28, *χ*^2^ (1) = 8.19, *p* = .004, *q* = .008
Finances	3.56 (0.94)	3.45 (0.91)	*β* = − 0.12, *χ*^2^ (1) = 3.25, *p* = .071, *q* = .101
Getting medication	3.54 (0.87)	3.25 (0.69)	*β* = − 0.35, *χ*^2^ (1) = 28.51, *p* < .001, *q* < .001
Getting food	3.92 (0.92)	3.48 (0.83)	*β* = − 0.46, *χ*^2^ (1) = 9.06, *p* = .003, *q* = .006
Your own safety/security	3.75 (0.97)	3.57 (0.81)	*β* = − 0.23, *χ*^2^ (1) = 4.93, *p* = .026, *q* = .048
Internet access	3.24 (0.76)	3.16 (0.64)	*β* = − 0.08, *χ*^2^ (1) = 0.46, *p* = .500, *q* = .571
Boredom	3.38 (1.06)	3.37 (0.94)	*β* = − 0.01, *χ*^2^ (1) = 0.01, *p* = .922, *q* = .962
Future plans	3.88 (1.06)	3.87 (0.94)	*β* = 0.02, *χ*^2^ (1) = 0.08, *p* = .774, *q* = .844
Over the past 2 weeks, have you been worried about the following			
Following the recommendations to prevent spread of COVID-19 correctly	3.03 (1.45)	2.83 (1.29)	*β* = − 0.14, *χ*^2^ (1) = 3.75, *p* = .053, *q* = .079
Catching COVID-19	3.09 (1.36)	3.01 (1.24)	*β* = − 0.10, *χ*^2^ (1) = 0.75, *p* = .388, *q* = .465
Friends or family catching COVID-19	3.82 (1.26)	3.82 (1.13)	*β* = − 0.00, *χ*^2^ (1) = 0.00, *p* = .974, *q* = .974

Autism group: *n* = 613, non-autism group: *n* = 431; Questions were rated on a five-point scale going from 1 (‘significantly less than usual’) to 5 (‘significantly more than usual’), except for the COVID-19 pandemic specific worries that were rated on a five-point scale going from 1 (‘not at all’) to 5 (‘very much so’). Participants were asked to indicate ‘not more than usual’ when not applicable

^a^Analysis only includes participants who indicated to have an pet, autism group *n* = 349, non-autism group *n* = 209

^b^Analysis only includes participants who indicated to be self-, part-time, or full-time employed, autism group *n* = 306, non-autism group *n* = 300

#### Impact of the pandemic on social contact and routines

Means and standard deviations of the COVID-19 pandemic-related changes on routines and social contact can be found in Table 4. Our LMMs revealed that the non-autism group more than the autism group felt the pandemic changed their social life, *β* = 0.53, *χ*^2^ (1) = 49.63, *p* < 0.001, *q* < 0.001. While the non-autism group hereby felt more socially isolated, *β* = 0.20, *χ*^2^ (1) = 4.70, *p* = 0.030, *q* = 0.048, the autism group felt more relieved from social stress, *β* = − 0.56, *χ*^2^ (1) = 22.46, *p* < 0.001, *q* < 0.001.

**Table 4:** Mean scores per group regarding the impact of the pandemic on topics related to the DSM criteria of Autism Spectrum Disorder

	Autism *M* (SD)	Non-autism *M* (SD)
*Social contact (domain I)*		
To what extent did the COVID-19 pandemic change your social life?	3.16 (1.41)	3.91 (1.17)
Due to the effects of the COVID-19 regulations on my everyday life, I feel socially isolated	2.59 (1.48)	2.89 (1.28)
Due to the effects of the COVID-19 regulations on my everyday life, I feel relieved from social stress	3.43 (1.43)	2.67 (1.30)
*Routines (domain II)*		
To what extent do you feel you have/had to adjust your daily routines due to the COVID-19 pandemic?	3.49 (1.27)	3.90 (1.08)
Due to the effects of the COVID-19 regulations on my everyday life, I feel stressed about changes to my daily routines	3.31 (1.39)	2.58 (1.24)
Due to the effects of the COVID-19 regulations on my everyday life, I enjoy the freedom to deviate from society’s expectations and to adjust my routines to my personal preference	3.46 (1.39)	3.16 (1.30)

Autism group: *n* = 613, non-autism group: *n* = 431; Questions were rated on a five-point scale going from 1 (‘Not at all’) to 5 (‘Very much so’)

The non-autism group more than the autism group felt they had to change their daily routines due to the pandemic, *β* = 0.32, *χ*^2^ (1) = 16.18, *p* < 0.001, *q* < 0.001. Nonetheless the autism group felt more stressed about the changes in daily routines than the non-autism group, *β* = − 0.58, *χ*^2^ (1) = 13.91, *p* < 0.001, *q* = 0.001. The groups did not differ in the extent they enjoyed the freedom to deviate from society’s expectations when adjusting their routines to the new situation, *β* = − 0.20, *χ*^2^ (1) = 2.75, *p* = 0.097, *q* = 0.129.

#### Impact of the pandemic on guidance and satisfaction with offered tips and tools

Of the participants from both groups who received guidance pre-pandemic from a qualified medical specialist (e.g. psychiatrist, psychologist, counsellor, thus entailing a broad range of services, practitioners and therapies), 46% indicated sessions being cancelled due to the COVID-19 pandemic (See Table 5). Across groups, for the majority of participants who had a wish for guidance, the wish was unrelated to the pandemic (58%). However, in the autism group, it was indicated more often that the wish for guidance was ‘both unrelated and related’ to the pandemic (42% versus 16%).

**Table 5:** Responses to questions related to help and guidance

	Autism *n* (%)	Non-autism *n* (%)
Did you receive any help/guidance from a qualified medical specialist (e.g. psychiatrist, psychologist, counsellor)?		
No, and I wouldn’t want to	142 (23.2)	277 (64.3)
No, but I would like to	98 (16.0)	55 (12.8)
Yes, but sessions are cancelled due to the COVID-19 pandemic	169 (27.6)	46 (10.7)
Yes, and sessions are continuing (face-to-face or online)	204 (33.3)	53 (12.3)
If indicated ‘no, but I would like to’:		
Is your wish to receive help/guidance related or unrelated to the pandemic		
Related	8 (8.2)	6 (10.9)
Unrelated	49 (50.0)	40 (72.7)
Both unrelated and related	41 (41.8)	9 (16.4)

Autism group: *n* = 613, non-autism group: *n* = 431

**Fig. 1:**
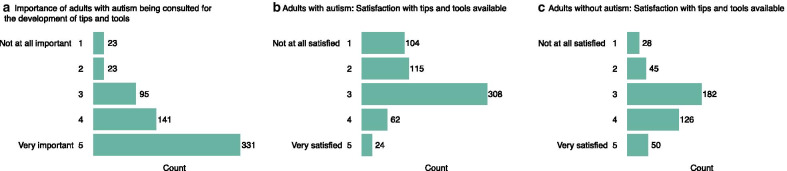
Responses on the tips and tools questions. *Note.* The *x*-axis represents the answering scale, the *y*-axis represents the number of participants that chose each response option. Full questions: (A) Many COVID-19 pandemic tips and tools are offered to the ASD population specifically, how important is it to you that individuals with ASD are consulted for the development of these tips and tools?; (B) How satisfied are you with the tips and tools that are available for individuals with ASD during the current COVID-19 pandemic?; (C) How satisfied are you with the tips and tools that are available for individuals with ASD during the current COVID-19 pandemic? Questions (A) and (B) were for the autism group only, whereas question (C) was for the non-autism group only

Figure 1a shows that the majority of adults with autism in our sample find it important that adults with autism are being consulted for the development of COVID-19 pandemic tips and tools. Furthermore, more adults with autism indicated to be unsatisfied (answering options 1 or 2: *n* = 219) than satisfied (answering options 4 or 5: *n* = 86) with the available tips and tools for individuals with autism during the COVID-19 pandemic (Fig. 1b). Moreover, less adults without autism indicated to be unsatisfied (answering options 1 or 2: *n* = 73) than satisfied (answering option 4 or 5: *n* = 176).

### Qualitative analyses

#### Measures that may help manage mental health during the pandemic

Themes identified from responses of adults with autism to the open question inquiring about their needs during the pandemic can be found in Table 6. Adults with autism indicated a need for (continued) professional support (e.g. medical, psychological, household support) that is both affordable and accessible. Many adults with autism that did not need regular support before, reported to need it during the pandemic but did not know how to access relevant services. Furthermore, many adults with autism lost some, if not all, of the support they received before the pandemic. The type of support that was lost varied widely, ranging from medical and psychological support to household support. Adults with autism that did report to receive support often indicated to prefer face-to-face support as soon as this was once again possible. Regarding online support, some adults with autism noted to experience anxiety on voice or video calls due to difficulties with the back-and-forth flow of social communication during calls, and would rather communicate via chat (i.e., text-based messaging). Nevertheless, there were some adults with autism that commended the accessibility of remote support. The open question further revealed that the need for help was often related to structuring their days (i.e., creating new routines that fit into pandemic life).

**Table 6:** Themes identified from responses of adults with autism to the following open question: What measures could be taken to help you manage your mental health during the current COVID-19 pandemic?

Theme	Subtheme	Example quote
(Continued) professional support		“I need to talk to my psychologist from time to time, but this is impossible right now as it is ‘not essential’” p. 38 “Would be nice if a professional checked up, helped with implementing tips and tools and some measure of reassurance was possible” p. 148 “Help with nuanced issues arising from the pandemic—changes to social codes, how to behave around others” p. 171
	Affordable and accessible	“More easily accessible and affordable psychological/therapeutic help (via Skype, chat …)” p. 318
	Availability of various communication mediums	“Something that would help me and many others would be if access to primary healthcare was available by some means other than making a phone call—e.g. SMS, online text chat, etc. Like a lot of autistic people, I have a lot of anxiety about making or receiving phone calls. If I need healthcare I have to agree to answer a phone call at an unspecified time. If I don't answer, no healthcare. This is very difficult to contemplate” p. 407
	Help structuring days	“Help with creating a routine to fit the pandemic and change.” p. 333
Clearer information and rules		“It would help me a lot if the rules were much clearer. It would avoid a lot of stress” [Translated] p. 942 “Difficulty in applying rules, because they are often ambiguous or too vague (what is still allowed, what is not). You think it's clear, and then someone else in the media brings up with his or her own opinion about those rules, making it a mess. I think that for me the extra stress that occurs from COVID-19 concerns social stress because of the new but unclear rules, and not stress by the virus itself” [Translated] p. 27
	Autism-tailored information and advice	“ASD Societies contacting individuals who are registered with them to advise them of the situation” p. 8 “The only tips and tricks that are easily available for people with ASD are catered towards children or people with ASD and learning disabilities” p. 224 “I was not aware that there were tips and tools for people with ASD specifically. Being made aware of these things might help.” p. 415

Both adults with and without autism expressed the need for clearer information and rules. However, whereas adults without autism mainly described the lack of clarity as an annoyance, adults with autism made explicit statements of distress. This lack of clarity and the accompanying distress led adults with autism to avoid activities that were still allowed, as they were afraid to break protocols (e.g., not going for walks alone or with members of their household at all, as it was not specified how far from their house they were allowed to go). Adults with autism further wished for (more) autism-specific information and advice that was specifically tailored to adults with autism, as many noted that existing information was mostly directed to parents of children with autism. The answers also indicated that the sources at that time were not yet promoted widely enough, as some were not aware of them.

#### COVID-19 pandemic-related changes that are the most difficult

The two themes identified from responses of adults with autism to the open question inquiring about COVID-19 pandemic-related difficulties can be found in Table 7. One theme related to a loss of routine. Many adults with autism indicated to have a clear daily or weekly routine they stuck to pre-pandemic, which they lost because of the pandemic affecting them negatively. They emphasized the importance of structure in their daily life to maintain their mental health, and the experienced difficulty with having to create a new routine by themselves. Some adults with autism also noted they missed social contact in their daily/weekly routines, overlapping with the second theme, namely, a loss of social contact. Both adults with and without autism mentioned an negative impact of the pandemic on their social life. However, whereas adults without autism mainly expressed the burden of missing their pre-pandemic social life (seeing others face-to-face), adults with autism rather described the burden of losing their social support network, which for some people led to full social isolation in an already stressful time. Some adults with autism also expressed the need for social contact to keep up their social skills. Furthermore, answers to this open question revealed that adults with autism experienced new social interaction difficulties directly related to the pandemic, such as not being able to read faces because of face masks, and having difficulty with the back-and-forth flow of social interaction during video calls.

**Table 7:** Themes identified from responses of adults with autism to the following open question: What changes, due to the effects of the COVID-19 regulations on your everyday life, are the most difficult for you (if any)?

Theme	Example quote
Loss of routine	“Usually I try to have five (sometimes short) live contacts every day, as otherwise I get lonely, which isn’t possible right now. I usually also have fixed events (library, movie, class, market,..) that give structure to my life, which I’ve also lost” [Translated] p. 1 “Every form of external structure to my days have disappeared. Since I don’t do well having to structure my day by myself, I’m not doing well with that” p. 610 “Knowing that I can’t go out even if I would normally choose not to is hard. It’s also hard not following normal routines and meeting with people I would normally. I had just started going to a group to help with my social interaction which has had to stop. It’s hard knowing I was making positive steps towards improving my social interactions and I’ve had to pause it. I know it is a needed measure to stop the virus but it’s still hard” p. 205 “Almost everything that was in my diary has fallen away. The first days after the measures were announced, and subsequently when they were being made stricter, I had to keep scratching things, until there was almost nothing left. All that unstructured time to be planned in again makes me very agitated.” [Translated] p. 14
Loss of social contact	“The stress of not being able to see loved ones outside the house and feeling crippled by social isolation, with no idea when it'll end. I feel a neurotypical person in this situation will have a vast number of people to interact with in varying ways. I have 2 who I'm in touch with outside my home and only 1 is via Skype. As an autistic I have a limited social circle and now it's even smaller, I worry about autistics who live alone consistently too.” p. 257 “The smallest social things I have managed to do so far (even if that is only one hug a month) from which I receive support, are now completely gone” [Translated] p. 25 “The lack of social and physical contact is debilitating. And the uncertainty about how long I have to keep this up takes me into a downward spiral” [translated] p. 2 “I’ve kind of liked having my own “sanctuary” at my house and being able to control my involvement with others but I am human, despite autism, and I am starting to miss the option of socializing” p. 977

Other changes that both groups found difficult were not being able to spend time alone due to living with others, balancing work-life while working from home, balancing work with taking care of children (including home-schooling), and the uncertainty of how long the pandemic and the associated changes will last.

#### COVID-19 pandemic-related changes that cause the most stress/anxiety

Themes identified from responses of adults with autism to the open question inquiring about COVID-19 pandemic-related stress/anxiety can be found in Table 8. By far the most often reported anxiety-provoking theme for adults with autism was related to shopping (grocery, but sometimes also for medication). The mentioned reasons for this were twofold. First, shopping routines were disrupted. This is due to, among other things, the unavailability of specific food and non-food products, the introduction of limits on time spent in the shop and on the number of products one may buy, and having to line up to enter the shop. Second, shopping was described as more stressful due to the social distancing rules. On the one hand, some adults with autism found it difficult to adhere to the rules themselves, on the other hand, being in an environment where other people do not always adhere to the rules induces anxiety and frustration.

**Table 8:** Themes identified from responses of adults with autism to the following open question: What changes, due to the effects of the COVID-19 regulations on your everyday life, have caused the most stress/anxiety (if any)?

Theme	Subtheme	Example quote
Groceries	Disrupted shopping routines	“The unavailability of my specific groceries, for weeks now. Getting to the shop and seeing empty shelves, yet again, bring me to tears. Because it’s something I have absolutely no control over. And having to change what I eat on top of everything else is just too much” p. 611 “Unable to do groceries due to meltdowns/ or panic attacks not sure which of these is the culprit. Sensory overload is entangled with supermarkets on a regular day, with all the change it’s become impossible to do on my own” p. 440
		“shopping—not being able to get my routine food” p. 86
	Social distancing rules	“Not being able to go grocery shopping without a lot of anxiety. I only go once a week, whereas I was used to go almost daily. I am very scared about people at the shops not adhering to the rules” p. 432 “Grocery shopping is awful now! It was always awful to begin with, but with all the extra regulations it’s even more stressful now. Constantly having to keep my distance and reminding myself is an extra, unwanted step in this already exhausting process of having to do everything right, having to think of everything. I now come home upset and sweaty every time.” p. 442
Going back to normal		“The sudden change in my routine and then the sudden change in going back to my previous routine when this has ended. My threshold for external stimulus is non-existence now so I am fearful that, when I do return to work, I am going to be exhausted due to sensory overload on my commute to/from work and the social interaction is going to also be exhausting. I am not "masking" anymore and I am concerned I will have forgotten how to do that and how to hide my stimming. The reality is it is probably muscle memory at this point in my life but the exhaustion from external stimuli is worrying me” p. 977 “The relief of not having to mask anymore, but simultaneously the worry I will not be able to join back the rat race as quickly as the others. When things will open back up, expectations of enjoying the social get together will be super high and I will be enormously stressed and out of practice in acting normal” p. 579 “After a few weeks I have now gotten used to this calm pace, these new social rules slowly seem clear to me, so now it gives me a little anxiety that afterwards I will have to get used to the hustle and bustle and chaos again, and having to find my own way in this.” [Translated] p. 317

Another recurrent anxiety-provoking theme was related to the prospect of having to go back to ‘normal’ after the pandemic. Adults with autism indicated to feel anxious about the prospect of having to change their routine yet again, possibly repeatedly, to adjust to the gradual lifting of lockdown and other pandemic-related measures. Furthermore, they feel anxious about the prospect of a sudden return of external stimuli (e.g. traffic, noise, more people being outside) paired with a loss of coping skills to deal with them. That is, many adults with autism protected themselves from sensory overload pre-pandemic by means of coping skills, which they often needed less of during lockdown, causing them to fear a loss of capability to cope with sensory overstimulation. Similarly, they fear to have lost their social skills, which makes the foreseen pressure to attend social events and return to a work/school environment with new social norms stress-inducing.

Other recurring topics for both adults with and without autism were worries about employment and finances, as well as worries about one’s own health and that of close others.

#### COVID-19 pandemic-related changes that made life more pleasant

Themes identified from responses of adults with autism to the open question inquiring about COVID-19 pandemic-related changes that made life more pleasant can be found in Table 9. One overarching theme was identified, as nearly all answers were related to changes that made the world a little more autism-friendly. Two subthemes further break down why. First, many adults with autism described a feeling of solidarity, now that their lives did not differ as much anymore from those of the people around them. For example, some adults with autism explained that, for once, the whole population had to socially isolate themselves for their own good—something that many adults with autism already have experience with, as they sometimes have to socially isolate themselves to reduce sensory overload. Second, lockdown reduced sensory and social overload. The outside world is quieter (i.e., less sensory stimulating), and many adults with autism described a feeling of relief from certain social stressors such as obligatory parties and appointments, spontaneous visits from others, or strangers getting too close.

**Table 9:** Themes identified from adults’ with autism responses to the following open question: What changes, due to the effects of the COVID-19 regulations on your everyday life, have made life more pleasant for you (if any)?

Theme	subtheme	Example quote
Autism friendly world	Social cohesion/ solidarity	“As the entire nation is on lockdown, and everyone is self-isolating, I feel like I am finally at one with society. My way of living life is the norm. I no longer have to feel alone. I have a sense of belonging that everyone is in the same situation and we're all in this together” p. 629 “I notice that I find the contact with friends and my parents more pleasant now: I feel like my own and their daily worlds have more of a common denominator now. There’s a difficult situation now, that others also intuitively experience as being difficult. (…) Furthermore, interactions are more frequent but shorter, maybe that’s why I feel less like I have to dig into my reserves in order to lead the social life that I want.” [Translated] p. 668
	Reduced sensory and social overload	“These times are in many respects more autism-friendly. There’s less car traffic and noise in my neighbourhood. There’s less traffic jam frustration and fewer exhaust gases. Working from home is a nice experiment that the crisis made possible. It is less stressful, because I am not disturbed and distracted by colleagues. And because I can take breaks whenever I need it, without worrying about social control. And the stress from socially obligated small talk is no longer there.” [Translated] p. 505
		“No more handshaking, no more people getting way too close and not feeling weird for staying at home all the time” p. 233

Recurring topics that both adults with and without autism mentioned were: having more time for themselves (adults with autism particularly seemed to enjoy the extra time they could spend on their special interests) and for family, having complete control over their daily structure (e.g., when to take a break from work, when to relax), working from home (although people also acknowledged that this would not work for months on end), and the overall pressure/ social stressors of everyday life being diminished.

Lastly, we would like to acknowledge two additional points that this open question revealed. First, although not specific to the COVID-19 pandemic, many adults with autism revealed that they received crucial support from within the autism community as they share their experiences with one another. Second, despite it being a small minority, it should be noted that there were some adults with autism for whom living conditions under the COVID-19 pandemic had actually improved overall, with the main reason for improvement being the reduced sensory and social overload.

## Discussion

This study investigated the psychological impact of the COVID-19 pandemic and the associated containment measures on adults with autism (as compared to adults without autism) across three countries. Specifically, we examined COVID-19 pandemic-related changes in mental health, issues related to the two autism symptom domains, satisfaction with pandemic-related information and tips, and participants’ wishes for guidance. For this, we used a mixed-method approach, combining quantitative and qualitative data. Our results highlight the burden of the pandemic on the mental health and daily life of adults with autism, and give insight into how they can be supported.

### Mental health

We found an increase in depression and anxiety symptoms in response to the pandemic for both the non-autism and the autism group. However, this increase in symptoms and the impact of these symptoms on everyday life was greater in the autism group. Moreover, an overstretched healthcare system paired with social distancing rules meant that many adults with autism lost some, if not all of the support they received before the pandemic, as evident from both our quantitative and qualitative results. These results are particularly worrisome considering that adults with autism ordinarily have higher rates of anxiety and depression than neurotypicals [18, 19], as was also reported by participants in our sample, and struggle to access mental health services [43, 44]. These results are in line with a recent report from the National Autism Society [27], where 9 out of 10 people with autism indicated to worry about their mental health during lockdown. We further found that the autism group worried significantly more than the non-autism group about their own safety/security, pets, work (even when they felt their job was safe), losing their job, and getting medication and food. The increase in worry concerning their pets in adults with autism is interesting as a previous pre-pandemic study by Dachez and Ndobo [45] found that some adults with autism seek support from pets as a coping strategy. The increase in work-related worries is concerning but not surprising, as autism was already associated with poor employment outcomes pre-pandemic [46]. Our quantitative results on getting medication and food were supported by our qualitative data. Shopping for groceries was by far the most often reported anxiety-provoking topic for adults with autism, as revealed by the qualitative findings, with some also particularly highlighting their worries about the reduced accessibility of medication. Shopping was described as more stressful due to the social distancing rules and disrupted shopping routines. To our knowledge, no study as of yet has specifically investigated everyday life topics of worries in autism, so it remains unknown whether adults with autism for instance also worried more about access to food or medication pre-pandemic. Research efforts aimed to learn about everyday life topics of worries in adults with autism would not only be worthwhile in light of our current results to determine if post-pandemic worries are different from those that increased during the pandemic, but may also add to the mood and anxiety literature on autism.

### Social life

Further, we found that the non-autism group, more than the autism group, felt the pandemic changed their social life, and consequently felt more socially isolated. These results may be explained by previous research showing that adults with autism were already more likely than neurotypicals to face loneliness and social isolation before the pandemic (e.g. [47, 48]), making the change to their social life caused by the pandemic less pronounced for them. Still, the difficulty most often reported by *both* groups was the loss of social contact. With autistic individuals already more likely than neurotypicals to face loneliness and social isolation, losing the access to their, usually already smaller (e.g., [47, 48]), support network appears to be a great burden on adults with autism. At the same time, we also found that the autism group felt more relieved from social stress compared to the non-autism group. This relief from certain social stressors was in line with our qualitative results. However, the qualitative results also brought to light that the pandemic came with new social challenges, such as difficulty reading facial expressions because of face masks.

### Routines

On average, the autism group felt more stressed than the non-autism group about the changes to their daily routines that the pandemic is causing. This finding was validated by our qualitative findings as many adults with autism mentioned the loss of routine to be one of the most difficult pandemic-related changes. Moreover, this finding is in accord with the diagnostic criteria (i.e., domain II) of autism (e.g., difficulty with transitions; [49]), and explains the voiced need for help with creating a new routine. Our qualitative findings further reveal that the stress induced by the disruption of routines also translates to prospective anxiety. That is, many adults with autism report experiencing anxiety due to the prospect of the ever-changing pandemic-related rules and the accompanying need to repeatedly adjust their routines.

### Implications

Our findings give important insights into how adults with autism can be supported in order to mitigate further mental health problems during the ongoing COVID-19 pandemic and possible future public health crises. Foremost, the majority of adults with autism find it important that they themselves are being consulted for the development of COVID-19 pandemic tips and tools. We therefore recommend collaborations with adults with autism in endeavors aimed at supporting them during the COVID-19 pandemic. Input from adults with autism may also increase satisfaction with the COVID-19 pandemic information and advice, and the tips and tools offered. COVID-19 pandemic information and advice should be clear and coherent, and widely promoted so that it reaches its target audience. Moreover, as our results indicate that most autism-specific information is directed towards parents with children with autism, we encourage efforts into the development of autism-specific information and advice specifically tailored to *adults* with autism.

In addition, ensuring continued, affordable, and accessible support at this time should be of the utmost priority. This is especially important given the negative impact of the pandemic on the mental health of the majority of adults with autism, and because the number of unmet formal support needs is found to be negatively associated with the quality of life of adults with autism [50]. The impact of the pandemic on for instance the social life and routines of adults with autism is inevitable. However, supporting them in the maintenance of a social network (which can be online as well), in creating alternative routines, and in updating those routines to the ongoing changes could soften the blow. Regarding social support, our results revealed that adults with autism received crucial support from within the autism community, which was previously identified as a coping strategy among adults with autism [45]. Therefore, stimulating adults with autism to engage in social contact within the autism community may be advisable.

When offering remote support, one should preferably provide various communication media as some adults with autism noted to experience anxiety on voice or video calls and therefore prefer text-based messaging. Anxiety experienced over voice or video calls in adults with autism was also previously found (see also for an elaborate discussion of the needs of adults with autism in video calling, [51]). Our results revealed that some adults with autism preferred face-to-face, whereas others commended the accessibility of remote online support. There is still abundant room for further research in determining for whom online healthcare may be beneficial, and how to provide effective remote mental health support to adults with autism during and after the COVID-19 pandemic [52].

Furthermore, to alleviate the anxiety experienced during shopping, one could advertise specific shopping times as autism-friendly, or a buddy system, pairing adults with autism with a buddy who can shop for them, could be introduced.

Lastly, we also found some COVID-19 pandemic-related changes that were experienced as pleasant, specifically the feeling of social solidarity, and reduced sensory and social overload. For some adults with autism, the reduction in sensory and social overload even led to overall improved living conditions during the COVID-19 pandemic. Although only the small minority of our autism sample preferred the COVID-19 pandemic living conditions, this group should not be overlooked when drawing conclusions about the best ways of taking action. On the contrary, in order to create a more inclusive society, we should learn from these positive experiences that emerged during the COVID-19 pandemic. It would be worthwhile for future research to investigate how we could promote continuity of social solidarity and reduced sensory and social overload post-pandemic.

### Strengths and limitations

To our knowledge, this study is the first to investigate the impact of the COVID-19 pandemic and the associated containment measures on adults with autism. Strengths of this study include the inclusion of a control group, the inclusion of multiple countries, the mixed-method approach of qualitative and quantitative data gathering, and the timing of the study, as it was conducted in the very first weeks of lockdown. As the pandemic undeniably has an impact on the whole population, the comparison with adults without autism enables us to gain insight into the specific impact of the pandemic on adults with autism. Data was gathered from three countries (Belgium, the Netherlands, and the UK). We controlled for country-related variation in the analyses and therefore our findings apply to adults with autism across these countries. Including qualitative data allowed us to gather more comprehensive data, to validate our quantitative findings, and to give a voice to the participants to ensure our findings are grounded in their experiences. Furthermore, the timing of the study gives unique insight into the experience of adults with autism in the very beginning of the pandemic.

The current study also has a number of limitations. First, as with many online survey studies, our sample is likely to reflect some degree of selection bias. Particularly, we expect some underrepresentation of extreme cases, that is, people that were either minimally or very affected by the pandemic. Second, the findings of this study are limited to the immediate experiences in the beginning of the pandemic, and are therefore restricted to the short-term effects of the pandemic. Future research should also examine the long-term effects of the pandemic, especially now that it is becoming more evident that the pandemic is likely to have a prolonged course. Third, generalizing our findings should be done with care. As mentioned, country of residence only explained a very small part of the variance (± 2.5%) in our study that included residents of Belgium, the Netherlands, and the UK. Still, the findings may not generalize to other countries in- or outside Europe. It is also worth mentioning that participants with Belgian residence were overrepresented in our sample in comparison to the other two countries. Our findings may also not be generalized to other subgroups of individuals with an autism diagnosis, such as children and individuals with intellectual disability. Future research could also focus on more narrow subgroups within the adult population, as we took a rather inclusive and broad approach (e.g., the age ranged between 18 and 81 years). It is important to note that although the groups did not differ on common matched-for variables such as age, gender ratio, and years of education (taken as a rough proxy for SES [53]), there were differences between groups on other variables such as pre-existing mental health difficulties and employment status, with adults reporting more mental health difficulties and lower employment status. However, these differences are representative of what is generally found in the autism population [18, 19, 46]. Moreover, this makes the greater pandemic-related increase in mental health problems as found for adults with autism even more worrisome. Lastly, we want to emphasize that inherent to group comparisons, the findings may not apply to all individuals in our sample as there is undoubtedly variability in how people with and without autism experience the pandemic.

## Conclusion

The results of our study highlight the burden of the pandemic on adults with autism and shed light on how to support them during this COVID-19 pandemic. The greater impact of the pandemic on the mental health of adults with autism emphasizes the need for accessible, affordable (continued) support from health services to manage their mental health. Guidance may focus on the maintenance of a social network, and adjusting routines to the rapid ongoing changes. Further, elevating shopping-related anxiety may help adults with autism cope during this pandemic. Lastly, there is a need for COVID-19 pandemic-related tips, tools and information tailored to adults with autism that are created in collaboration with them. We hope that the findings of this study will increase awareness about the negative impact of the COVID-19 pandemic on adults with autism, and that our recommendations will be translated by the relevant institutions into targeted, helpful strategies.

## Data Availability

The dataset analysed during the current study is not publicly available to ensure that individual privacy of these sensitive data cannot be compromised, but is available from the corresponding author on reasonable request.

## Appendix A

**A. ASD**

**Table:**

A1. Do you have a formal clinical diagnosis of ASD? This includes ASD (DSM-5) as well as diagnoses based on earlier DSM versions, such as Autistic Disorder, Asperger's disorder, and Pervasive Developmental Disorder—Not Otherwise Specified. Yes/No	
A1a. Please specify which diagnosis:	Autism Spectrum Disorder
	Asperger’s Syndrome
	Autistic Disorder
	Pervasive Development Disorder –NOS
	Other:
A1b. At what age did you receive the diagnosis?	(number list)
A2. Do you have a formal clinical diagnosis of any of the following (tick any that apply):	
	Attention Deficit/Hyperactivity Disorder
	a depressive disorder
	an anxiety disorder
	other, specify
	none

**B. Demographics**

**Table:**

B1. Your age	
B2. Your biological sex	male
	female
	intersex
	other/prefer not to say
B3. Your employment status	student in high school
	student at university
	self-employed
	in part-time employment
	in full-time employment
	unable to work due to disability
	homemaker/full-time parent
	unemployed and seeking work
	retired
B4. Years of education, starting form year 1 (learning to read)	
Note: if you’re a student and you’re currently in your second term, please include this academic year	
*Note: in case you aren't sure, please respond with your best estimation*	
B5. Country of residence	
	Belgium
	the Netherlands
	the UK

**C. COVID-19 specific**

**Table:**

C1. Have you had COVID-19?	Yes: diagnosed and recovered
	Yes: diagnosed and still ill
	not formally diagnosed but I suspect to
	have COVID-19
	No
C2. How much time on average do you spend actively looking up/ reading about COVID-19-related information every day?	
	< 1 h
	1–2 h
	3–4 h
	≥ 5 h
C3. How much time on average do you spend going over COVID-19-related information in your mind every day?	
	< 1 h
	1–2 h
	3–4 h
	≥ 5 h
C4. How would you rate your knowledge level on the COVID-19 virus and situation?	
	1—very poor knowledge
	to
	5—very good knowledge
C5. Are you following the recommendations from national authorities to prevent spread of COVID-19?	
	1—not at all
	to
	5—very much so
C6. How has COVID-19 influenced your daily routine? (tick any that apply)	
Note: by 'self-isolating' we mean staying at home and avoiding contact with people outside the household. If you have symptoms you may also be avoiding contact with people within your household	
Note: by 'social distancing' we mean staying far enough away from people outside the household so that the virus cannot spread from one person to another. 'Far enough' is defined as 2 m (6ft) in the UK and 1.5 m in Belgium and the Netherlands	
	- I am living my life as normal
	- I am not self-isolating, but I have stopped going to work like normal and started working from home
	- I am not self-isolating specifically, but I have cut down on my usual activities as a precaution/I am social distancing
	- I am self-isolating due to a diagnosis of COVID-19 or possible symptoms
	- I am self-isolating because I have an existing medical condition or am categorized as high risk
	- I am self-isolating as I am worried about spreading the virus to others or getting ill (but I am not high risk)
	- I am self-isolating to protect a family member, friend or housemate who has an existing medical condition/is high risk
	- I am self-isolating as it has been advised/ordered by the government or local authorities
	- I am self-isolating but this is NOT because of COVID-19 but because of another reason (e.g. a pre-existing health condition or disability)
C7. In the past 7 days how many days have you:	
7a been self-isolating (not leaving the house)	
7b been outside for 15 min or longer	
7c been outside to meet someone for 15 min or longer	
7d had face-to-face contact with someone I live with for 15 min or longer	
7e had face-to-face contact with another person for 15 min or longer (excluding someone you live with)	
7f had a phone or video call with another person for 15 min or longer	

**D. Social contact**

**Table:**

D1. What is your living situation?	Living alone
	Living with partner and/or children
	Living with parents
	Living with flatmates
	Assisted living facility
D2. How many adults are living with you in the household? (Note: do not count yourself)	
D2a if you have a partner living with you: what is his/her employment status at the moment?	
	at school
	at university
	self-employed
	in part-time employment
	in full-time employment
	unable to work due to disability
	homemaker/full-time parent
	unemployed and seeking work
	retired
	I don’t live with a partner
D3. How many children are living with you in the household?	
D3a Please specify below the age(s) of your child(ren) currently living with you	
D4. Do you have any of the following pets (tick any that apply)	no pets
	cat
	dog
	Other, specify
D5. Usually in your life, how often do you meet up with people face to face socially, not for work (e.g. friends, family, relatives or social events with colleagues). This does not include the people you live with	
	Everyday
	Three or more times a week
	Once or twice a week
	Once or twice a month
	Less than once a month

**E. AQ-short**

**Table:**

E1. Choose one response that best describes how strongly each item applies to you
1. I prefer to do things with others rather than on my own
2. I prefer to do things the same way over and over again
3. If I try to imagine something, I find it very easy to create a picture in my mind
4. I frequently get so strongly absorbed in one thing that I lose sight of other things
5. I usually notice car number plates or similar strings of information
6. When I’m reading a story, I can easily imagine what the characters might look like
7. I am fascinated by dates
8. In a social group, I can easily keep track of several different people’s conversations
9. I find social situations easy
10. I would rather go to a library than to a party
11. I find making up stories easy
12. I find myself drawn more strongly to people than to things
13. I am fascinated by numbers
14. When I’m reading a story, I find it difficult to work out the characters’ intentions
15. I find it hard to make new friends
16. I notice patterns in things all the time
17. It does not upset me if my daily routine is disturbed
18. I find it easy to do more than one thing at once
19. I enjoy doing things spontaneously
20. I find it easy to work out what someone is thinking or feeling just by looking at their face
21. If there is an interruption, I can switch back to what I was doing very quickly
22. I like to collect information about categories of things (e.g., types of cars, birds, trains, plants)
23. I find it difficult to imagine what it would be like to be someone else
24. I enjoy social occasions
25. I find it difficult to work out people’s intentions
26. New situations make me anxious
27. I enjoy meeting new people
28. I find it very easy to play games with children that involve pretending

**F. COVID-19 × ASD DSM criteria**

**Table:**

F1. Choose one response that best describes how much each statement applies to you
(Five-point Likert scale ranging from: 1 Not at all–5 very much so)
F1a. To what extent do you feel you have/had to adjust your daily routines due to the COVID-19 pandemic?
F1b. Due to the effects of the COVID-19 regulations on my everyday life, I feel stressed about changes to my daily routines
F1c. Due to the effects of the COVID-19 regulations on my everyday life, I enjoy the freedom to deviate from society’s expectations and to adjust my routines to my personal preference
F2. Choose one response that best describes how much each statement applies to you
(Five-point Likert scale ranging from: 1 Not at all–5 very much so)
F2a To what extent did the COVID-19 pandemic change your social life?
F2b. Due to the effects of the COVID-19 regulations on my everyday life, I feel socially isolated
F2c. Due to the effects of the COVID-19 regulations on my everyday life, I feel relieved from social stress
F3. Over the past 2 weeks, have you been more or less worried about the following:
Note: In case not applicable choose ‘not more than usual’
Scale: significantly less than usual—slightly less than usual—not more than usual- slightly more than usual—significantly more than usual
F3a. marriage or other romantic relationship(s)
F3b. your pet
F3c. work (even if you feel your job is safe)
F3d. losing your job
F3e. finances
F3f. getting medication
F3g. getting food
F3h. your own safety/security
F3i. internet access
F3j. boredom
F3k. future plans
F4. Over the past 2 weeks, have you been worried about the following?
(Five-point Likert scale ranging from: 1 Not at all–5 very much so)
F4a. following the recommendations to prevent spread of COVID-19 correctly
F4b. catching COVID-19
F4c. friends or family catching COVID-19

**G. Patient health questionnaire (PHQ)**

**Table:**

Over the last 2 weeks, how often have you been bothered by any of the following?	
Scale: significantly less than usual—slightly less than usual—not more than usual- slightly more than usual—significantly more than usual	
1. Little interest or pleasure doing things	
2. Feeling down, depressed, or hopeless	
3. Trouble falling or staying asleep, or sleeping too much	
4. Feeling tired or having little energy	
5. Poor appetite or overeating	
6. Feeling bad about yourself—or that you are a failure or have let yourself or your family down	
7. Trouble concentrating on things, such as reading the newspaper or watching television	
8. Moving or speaking so slowly that other people could have noticed. Or the opposite—being so fidgety or restless that you have been moving around a lot more than usual	
9. Thoughts that you would be better off dead, or of hurting yourself	
G2. If you checked off an increase in any problems, how difficult have these problems made it for you to do your work, take care of things at home, or get along with other people?	
	Not difficult at all
	Somewhat difficult
	Very difficult
	Extremely difficult

**H. Generalized anxiety disorder 7-items (GAD-7) scale**

**Table:**

Over the last 2 weeks, how often have you been bothered by the following problems?
Scale: significantly less than usual—slightly less than usual—Not more than usual- slightly more than usual—significantly more than usual
1. Feeling nervous, anxious, or on edge
2. Not being able to stop or control worrying
3. Worrying too much about different things
4. Trouble relaxing
5. Being so restless that it's hard to sit still
6. Becoming easily annoyed or irritable
7. Feeling afraid as if something awful might happen
H2. If you checked off an increase in any problems, how difficult have these made it for you to do your work, take care of things at home, or get along with other people?

**I. Covid × ASD: guidance**

**Table:**

I1. In the last 6 months, did you receive any help/guidance from a qualified medical specialist	
(e.g. psychiatrist, psychologist, counselor)?	
	No, and I wouldn’t want to
	No, but I would like to
	Yes, but sessions are cancelled due to COVID-19 epidemic
	Yes, and sessions are continuing (face-to face or online)
I1a. IF indicated ‘No, but I would like to’:	
Is your wish to receive help/guidance related or unrelated to the pandemic:	
	related
	unrelated
	both unrelated and related
I2ASD. Many COVID-19 pandemic tips and tools are offered to the ASD population specifically, how important is it to you that individuals with ASD are consulted for the development of these tips and tools?	
1. Not at all important–5. Very important	
I3ASD. How satisfied are you with the tips and tools that are available for individuals with ASD during the current COVID-19 pandemic?	
1. Not at all satisfied–5. Very satisfied	
I3Control. Many COVID-19 pandemic tips and tools are offered. How satisfied are you with the tips and tools that are available during the current COVID-19 pandemic?	
1. Not at all satisfied–5. Very satisfied	

**Open questions**

1. What measures could be taken to help you manage your mental health during the current COVID-19 pandemic?
2. What changes, due to the effects of the COVID-19 regulations on your everyday life, are the most difficult for you (if any)?
3. What changes, due to the effects of the COVID-19 regulations on your everyday life, have caused the most stress/anxiety (if any)?
4. What changes, due to the effects of the COVID-19 regulations on your everyday life, have made life more pleasant for you (if any)?

## Appendix B: Pandemic-specific sample characteristics

See Table 10.

**Table 10:** Sample characteristics per group: COVID-19 pandemic specific and social situation during the pandemic

	Autism *M* (SD)/*n* (%)	Non-autism *M* (SD)/*n* (%)
Have you had COVID-19?^a^		
Yes	2 (0.3%)	5 (1.2%)
Not formally diagnosed but I suspect to have (had) COVID-19	107 (17.5%)	74 (17.2%)
No	504 (82.2%)	352 (81.7%)
How much time on average do you spend actively looking up/ reading about COVID-19-related information every day?		
< 1 h	316 (51.5%)	254 (58.9%)
1–2 h	223 (36.4%)	148 (34.3%)
3-4hours	50 (8.2%)	22 (5.1%)
≥ 5 h	24 (3.9%)	7 (1.6%)
How much time on average do you spend going over COVID-19-related information in your mind every day?		
< 1 h	218 (35.6%)	173 (40.1%)
1–2 h	191 (31.2%)	168 (39.0%)
3–4 h	124 (20.2%)	62 (14.4%)
≥ 5 h	80 (13.1%)	28 (6.5%)
How would you rate your knowledge level on the COVID-19 virus and situation?^b^	3.77 (0.92)	3.57 (0.86)
Are you following the recommendations from national authorities to prevent spread of COVID-19?^c^	4.56 (0.73)	4.59 (0.67)
In the past 7 days how many days have you:		
Been self-isolating (not leaving the house)	3.49 (2.64)	3.15 (2.59)
Been outside for 15 min or longer	3.88 (2.51)	4.32 (2.40)
Been outside to meet someone for 15 min or longer	0.80 (1.66)	0.75 (1.45)
Had face-to-face contact with someone I live with for 15 min or longer	4.46 (3.26)	5.50 (2.75)
Had face-to-face contact with another person for 15 min or longer (excluding someone you live with)	1.18 (1.96)	1.19 (1.80)
Had a phone or video call with another person for 15 min or longer	2.72 (2.29)	3.81 (2.24)
Usually in life, how often do you meet up with people face to face socially?		
Every day	40 (6.5%)	74 (17.2%)
Three or more times a week	123 (20.1%)	150 (34.8%)
Once or twice a week	211 (34.4%)	140 (32.5%)
Once or twice a month	132 (21.5%)	47 (10.9%)
Less than once a month	107 (17.5%)	20 (4.6%)
What is your living situation?		
Living alone	180 (29.4%)	53 (12.3%)
Living with partner and/or child(ren)	291 (47.5%)	270 (62.6%)
Living with parents	116 (18.9%)	64 (14.8%)
Living with flatmates	22 (3.6%)	44 (10.2%)
Assisted living facility	4 (0.7%)	0 (0.0%)
How many adults are living with you in the household? (Note: do not count yourself)	1.17 (1.13)	1.42 (1.03)
How many children are living with you in the household?	0.52 (0.96)	0.53 (0.93)
Do you have any pets? (% yes)	348 (56.8%)	209 (48.5%)

Autism group: *n* = 613, non-autism group: *n* = 431;

^a^We did not exclude participants on the basis of their response to this question, as all pandemic experiences were considered equally valuable

^b^Question was rated on a 5 point scale going from 1 (‘very poor knowledge’) to 5 (‘very good knowledge’)

^c^Question was rated on a 5 point scale going from 1 (‘not at all’) to 5 (‘very much so’)

## Footnotes

1 We use an abbreviated version of the diagnostic term: person with or without (a diagnosis) of autism (spectrum disorder). With this, we do not intend to take a stance in the ongoing person-first versus identity-first debate. We acknowledge and respect different preferences for language used to refer to a person with a diagnosis of ASD.

2 These three countries were included specifically as the authors had access to an autism population and/or connections in each of these countries for recruitment.

3 This included ASD (DSM-5) as well as diagnoses based on earlier DSM versions, such as Autistic Disorder, Asperger's disorder, and Pervasive Developmental Disorder—Not Otherwise Specified.

4 We report estimated standardized regression coefficients for all our LMMs as effect size (i.e. *β*). Note that across the LMMs only ± 2.5% of the variance could be attributed to the random effects of country.

## References

[1] WHO Director-General’s opening remarks at the media briefing on COVID-19—11 March 2020. [cited 2020 Oct 26]. https://www.who.int/director-general/speeches/detail/who-director-general-s-opening-remarks-at-the-media-briefing-on-covid-19---11-march-2020.

[2] Mertens G, Gerritsen L, Duijndam S, Salemink E, Engelhard IM (2020). Fear of the coronavirus (COVID-19): predictors in an online study conducted in March 2020. J Anxiety Disord.

[3] Greco V, Roger D (2003). Uncertainty, stress, and health. Pers Individ Dif.

[4] Southwick SM, Vythilingam M, Charney DS (2005). The psychobiology of depression and resilience to stress: implications for prevention and treatment. Annu Rev Clin Psychol.

[5] Holmes EA, O’Connor RC, Perry VH, Tracey I, Wessely S, Arseneault L (2020). Multidisciplinary research priorities for the COVID-19 pandemic: a call for action for mental health science. Lancet Psychiatry.

[6] Taylor MR, Agho KE, Stevens GJ, Raphael B (2008). Factors influencing psychological distress during a disease epidemic: data from Australia’s first outbreak of equine influenza. BMC Public Health.

[7] Main A, Zhou Q, Ma Y, Luecken LJ, Liu X (2011). Relations of sars-related stressors and coping to Chinese college students’ psychological adjustment during the 2003 Beijing sars epidemic. J Couns Psychol.

[8] Mihashi M, Otsubo Y, Yinjuan X, Nagatomi K, Hoshiko M, Ishitake T (2009). Predictive factors of psychological disorder development during recovery following SARS outbreak. Health Psychol.

[9] Wheaton MG, Abramowitz JS, Berman NC, Fabricant LE, Olatunji BO (2012). Psychological predictors of anxiety in response to the H1N1 (swine flu) pandemic. Cognit Ther Res.

[10] Cao W, Fang Z, Hou G, Han M, Xu X, Dong J (2020). The psychological impact of the COVID-19 epidemic on college students in China. Psychiatry Res.

[11] Cooke JE, Eirich R, Racine N, Madigan S (2020). Prevalence of posttraumatic and general psychological stress during COVID-19: a rapid review and meta-analysis. Psychiatry Res.

[12] Gonçalves AP, Zuanazzi AC, Salvador AP, Jaloto A, Pianowski G, Carvalho LF (2020). Preliminary findings on the associations between mental health indicators and social isolation during the COVID-19 pandemic. Arch Psychiatry Psychother.

[13] Smith L, Jacob L, Yakkundi A, McDermott D, Armstrong NC, Barnett Y (2020). Correlates of symptoms of anxiety and depression and mental wellbeing associated with COVID-19: a cross-sectional study of UK-based respondents. Psychiatry Res.

[14] Tull MT, Edmonds KA, Scamaldo KM, Richmond JR, Rose JP, Gratz KL (2020). Psychological outcomes associated with stay-at-home orders and the perceived impact of COVID-19 on daily life. Psychiatry Res.

[15] Wang C, Pan R, Wan X, Tan Y, Xu L, Ho CS (2020). Immediate psychological responses and associated factors during the initial stage of the 2019 coronavirus disease (COVID-19) epidemic among the general population in China. Int J Environ Res Public Health.

[16] Zhang SX, Wang Y, Rauch A, Wei F (2020). Unprecedented disruption of lives and work: Health, distress and life satisfaction of working adults in China one month into the COVID-19 outbreak. Psychiatry Res.

[17] Elsabbagh M, Divan G, Koh YJ, Kim YS, Kauchali S, Marcín C (2012). Global prevalence of autism and other pervasive developmental disorders. Autism Res.

[18] Kirsch AC, Huebner ARS, Mehta SQ, Howie FR, Weaver AL, Myers SM (2020). Association of comorbid mood and anxiety disorders with autism spectrum disorder. JAMA Pediatr.

[19] Lever AG, Geurts HM (2016). Psychiatric co-occurring symptoms and disorders in young, middle-aged, and older adults with autism spectrum disorder. J Autism Dev Disord.

[20] Gillott A, Standen PJ (2007). Levels of anxiety and sources of stress in adults with autism. J Intellect Disabil.

[21] Robertson AE, Stanfield AC, Watt J, Barry F, Day M, Cormack M (2018). The experience and impact of anxiety in autistic adults: a thematic analysis. Res Autism Spectr Disord.

[22] Ameis SH, Lai MC, Mulsant BH, Szatmari P (2020). Coping, fostering resilience, and driving care innovation for autistic people and their families during the COVID-19 pandemic and beyond. Mol Autism.

[23] Asbury K, Fox L, Deniz E, Code A, Toseeb U. How is COVID-19 affecting the mental health of children with special educational needs and disabilities and their families? J Autism Dev Disord. 2020; 1. 10.1007/s10803-020-04577-2 PMC7393330 32737668

[24] White LC, Law JK, Daniels AM, Toroney J, Vernoia B, Xiao S, et al. Brief report: impact of COVID-19 on individuals with ASD and their caregivers: a perspective from the SPARK cohort. J Autism Dev Disord. 2021; 1–8. 10.1007/s10803-020-04816-6 PMC7775834 33387233

[25] Eshraghi AA, Li C, Alessandri M, Messinger DS, Eshraghi RS, Mittal R (2020). COVID-19: overcoming the challenges faced by individuals with autism and their families. Lancet Psychiatry.

[26] Amorim R, Catarino S, Miragaia P, Ferreras C, Viana V, Guardiano M (2020). The impact of COVID-19 on children with autism spectrum disorder. Rev Neurol.

[27] National Autistic Society. Left stranded: The impact of coronavirus on autistic people and their families in the UK. 2020 [cited 2020 Oct 26]. https://s4.chorus-mk.thirdlight.com/file/1573224908/63117952292/width=-1/height=-1/format=-1/fit=scale/t=444295/e=never/k=da5c189a/LeftStrandedReport.pdf

[28] Mutluer T, Doenyas C, Aslan GH (2020). Behavioral implications of the Covid-19 process for autism spectrum disorder, and individuals’ comprehension of and reactions to the pandemic conditions. Front Psychiatry.

[29] Colizzi M, Sironi E, Antonini F, Ciceri ML, Bovo C, Zoccante L (2020). Psychosocial and behavioral impact of COVID-19 in autism spectrum disorder: an online parent survey. Brain Sci.

[30] Hoekstra RA, Vinkhuyzen AAE, Wheelwright S, Bartels M, Boomsma DI, Baron-Cohen S (2011). The construction and validation of an abridged version of the autism-spectrum quotient (AQ-short). J Autism Dev Disord.

[31] Kroenke K, Spitzer RL (2002). The PHQ-9: a new depression diagnostic and severity measure. Psychiatr Ann.

[32] Spitzer RL, Kroenke K, Williams JBW, Löwe B (2006). A brief measure for assessing generalized anxiety disorder: the GAD-7. Arch Intern Med.

[33] Bates D, Mächler M, Bolker BM, Walker SC (2015). Fitting linear mixed-effects models using lme4. J Stat Softw.

[34] Benjamini Y, Hochberg Y (1995). Controlling the false discovery rate: a practical and powerful approach to multiple testing. J R Stat Soc Ser B.

[35] Storey JD (2003). The positive false discovery rate: A Bayesian interpretation and the q-value. Ann Stat.

[36] Braun V, Clarke V (2006). Using thematic analysis in psychology. Qual Res Psychol.

[37] Loomes R, Hull L, Mandy WPL (2017). What is the male-to-female ratio in autism spectrum disorder? A systematic review and meta-analysis. J Am Acad Child Adolesc Psychiatry.

[38] Arnold S, Foley KR, Hwang YI, Richdale AL, Uljarevic M, Lawson LP (2019). Cohort profile: The Australian Longitudinal Study of Adults with Autism (ALSAA). BMJ Open.

[39] Bury SM, Jellett R, Spoor JR, Hedley D. “It Defines Who I Am” or “It’s Something I Have”: What Language Do [Autistic] Australian Adults [on the Autism Spectrum] Prefer? J Autism Dev Disord. 2020; 1–11. 10.1007/s10803-020-04425-3 32112234

[40] Kenny L, Hattersley C, Molins B, Buckley C, Povey C, Pellicano E (2016). Which terms should be used to describe autism? Perspectives from the UK autism community. Autism.

[41] Nederlandse vereninging voor Autisme. Voor een autismevriendelijke maatschappij. 2019 [cited 2020 Oct 26]. https://www.autisme.nl/wp-content/uploads/2019/02/NVA_corporate-brochure_7feb2019_LR.pdf

[42] Learning disability today. Government pledges to monitor autism employment gap | LDT. 2019 [cited 2020 Oct 26]. https://www.learningdisabilitytoday.co.uk/government-pledges-to-monitor-autism-employment-gap-for-the-first-time

[43] Camm-Crosbie L, Bradley L, Shaw R, Baron-Cohen S, Cassidy S (2019). ‘People like me don’t get support’: autistic adults’ experiences of support and treatment for mental health difficulties, self-injury and suicidality. Autism.

[44] Maddox BB, Gaus VL (2019). Community mental health services for autistic adults: good news and bad news. Autism Adulthood.

[45] Dachez J, Ndobo A (2018). Coping strategies of adults with high-functioning autism: a qualitative analysis. J Adult Dev.

[46] Chen JL, Leader G, Sung C, Leahy M (2015). Trends in employment for individuals with autism spectrum disorder: a review of the research literature. Rev J Autism Dev Disorders.

[47] Orsmond GI, Shattuck PT, Cooper BP, Sterzing PR, Anderson KA (2013). Social participation among young adults with an autism spectrum disorder. J Autism Dev Disord.

[48] van Asselt-Goverts AE, Embregts PJCM, Hendriks AHC, Wegman KM, Teunisse JP (2015). Do social networks differ? Comparison of the social networks of people with intellectual disabilities, people with autism spectrum disorders and other people living in the community. J Autism Dev Disord.

[49] American Psychiatric Association. DSM-5 Diagnostic Classification. In: Diagnostic and statistical manual of mental disorders. 2013.

[50] Renty J, Roeyers H (2006). Quality of life in high-functioning adults with autism spectrum disorder: the predictive value of disability and support characteristics. Autism.

[51] Zolyomi A, Begel A, Waldern JF, Tang J, Barnett M, Cutrell E (2019). Managing stress: the needs of autistic adults in video calling. Proc ACM Human Comput Interact..

[52] Harper G, Kenny L, Smith E, Bell A, Absoud M, Oomen D, et al. Impact of COVID-19 on autistic people: action briefing August 2020. [cited 2021 Jan 16]. www.autistica.org.uk/refs-covid

[53] Joseph RM, O’Shea TM, Allred EN, Heeren T, Kuban KK (2018). Maternal educational status at birth, maternal educational advancement, and neurocognitive outcomes at age 10 years among children born extremely preterm. Pediatr Res.

